# Supplementary figures and images for: Evaluation of pregnancy associated glycoproteins assays for on farm determination of pregnancy status in beef cattle
Source: PLoS One. 2024 Jul 25;19(7):e0306325. doi: 10.1371/journal.pone.0306325 (PMC11271854; doi:10.1371/journal.pone.0306325)

.


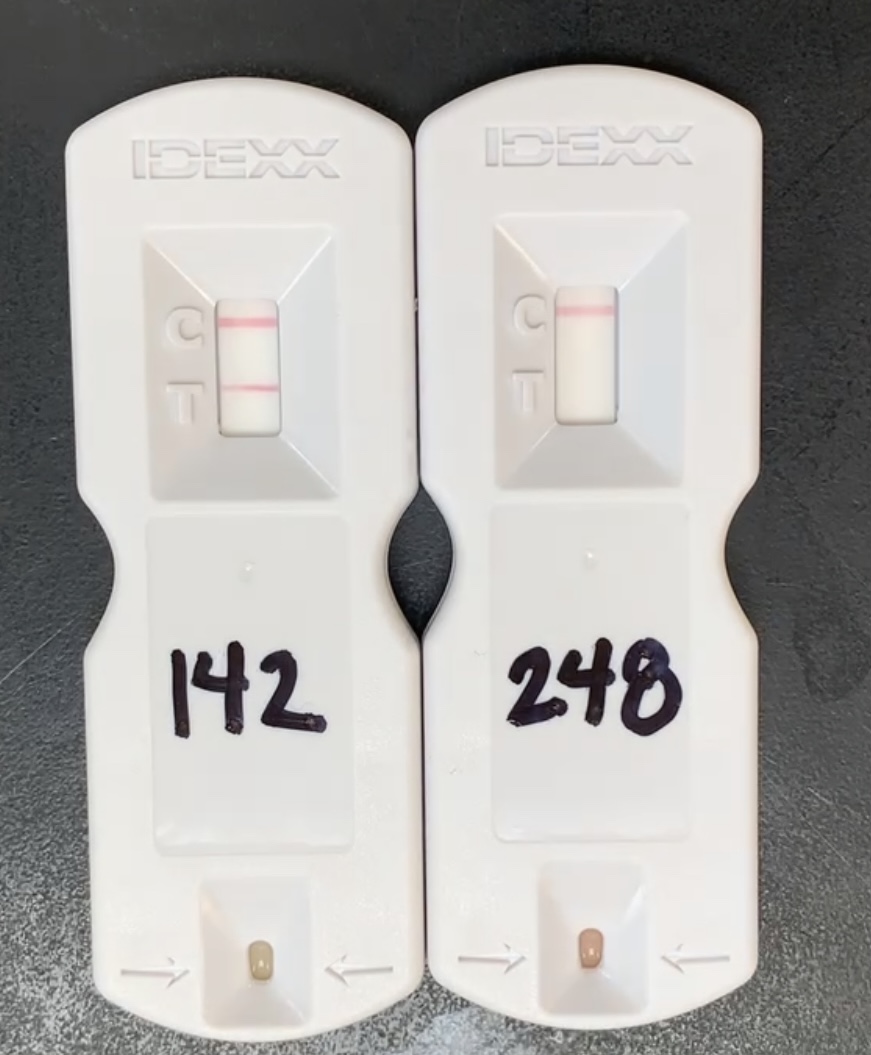

Supplement: S1 Fig — The test on the left indicates that particular female at the timepoint the test was taken is pregnant due to the visibility of the test sample, “T”, line. The test on the right indicates that particular female is not pregnant due to there only being one line visible and that is the internal positive control, “C”, line. If the C line does not show up at all the test is invalid and the sample should be reran on a different test. (DOCX) [file pone.0306325.s001.docx]
