# Supplementary material for: Evaluation of pregnancy associated glycoproteins assays for on farm determination of pregnancy status in beef cattle
Source: PLoS One. 2024 Jul 25;19(7):e0306325. doi: 10.1371/journal.pone.0306325 (PMC11271854; doi:10.1371/journal.pone.0306325)
Supplement: S2 File — This is the S2 data file. (DOCX) [file pone.0306325.s003.docx]

| herd | ID | age | day | day2 | pag | lateral | RV |
| --- | --- | --- | --- | --- | --- | --- | --- |
| 1 | 105 | cows | 8 | 14 | 3.6265 | P | P |
| 1 | 105 | cows | 16 | 21 | 3.1865 | P | P |
| 1 | 105 | cows | 22 | 28 | 1.6255 | P | P |
| 1 | 105 | cows | 29 | 35 | 1.1835 | P | P |
| 1 | 105 | cows | 36 | 42 | 0.5265 | P | O |
| 1 | 105 | cows | 43 | 49 | 0.3075 | P | O |
| 1 | 105 | cows | 50 | 56 | 0.1875 | P | O |
| 1 | 105 | cows | 57 | 63 | 0.117 | P | O |
| 1 | 105 | cows | 64 | 70 | 0.0615 | O | O |
| 1 | 105 | cows | 71 | 77 | 0.048 | P | O |
| 1 | 139 | cows | 3 | 7 | 3.6065 | P | P |
| 1 | 139 | cows | 10 | 14 | 3.0975 | P | P |
| 1 | 139 | cows | 17 | 21 | 1.6555 | P | P |
| 1 | 139 | cows | 24 | 28 | 1.131 | P | P |
| 1 | 139 | cows | 31 | 35 | 0.707 | P | P |
| 1 | 139 | cows | 40 | 42 | 0.2025 | P | P |
| 1 | 139 | cows | 52 | 56 | 0.1585 | P | O |
| 1 | 139 | cows | 59 | 63 | 0.088 | P | O |
| 1 | 142 | cows | 4 | 7 | 3.5165 | P | P |
| 1 | 142 | cows | 11 | 14 | 2.649 | P | P |
| 1 | 142 | cows | 18 | 21 | 1.1375 | P | P |
| 1 | 142 | cows | 27 | 28 | 0.7255 | P | P |
| 1 | 142 | cows | 39 | 42 | 0.2995 | P | O |
| 1 | 142 | cows | 46 | 49 | 0.166 | P | O |
| 1 | 148 | cows | 4 | 7 | 3.7765 | P | P |
| 1 | 148 | cows | 10 | 14 | 2.924 | P | P |
| 1 | 148 | cows | 17 | 21 | 2.4225 | P | P |
| 1 | 148 | cows | 24 | 28 | 0.8805 | P | P |
| 1 | 148 | cows | 31 | 35 | 0.6135 | P | P |
| 1 | 148 | cows | 38 | 42 | 0.2685 | P | P |
| 1 | 148 | cows | 45 | 49 | 0.13 | P | P |
| 1 | 148 | cows | 52 | 56 | 0.0945 | P | O |
| 1 | 148 | cows | 59 | 63 | 0.041 | P | P |
| 1 | 148 | cows | 68 | 70 | 0.0365 | O | O |
| 1 | 196 | cows | 4 | 7 | 3.2305 | P | P |
| 1 | 196 | cows | 11 | 14 | 2.077 | P | P |
| 1 | 196 | cows | 18 | 21 | 1.805 | P | P |
| 1 | 196 | cows | 25 | 28 | 1.086 | P | P |
| 1 | 196 | cows | 32 | 35 | 0.264 | P | P |
| 1 | 196 | cows | 41 | 42 | 0.174 | P | O |
| 1 | 196 | cows | 50 | 56 | 0.095 | O | O |
| 1 | 196 | cows | 60 | 63 | 0.042 | O | O |
| 1 | 224 | cows | 5 | 7 | 3.237 | P | P |
| 1 | 224 | cows | 12 | 14 | 1.9415 | P | P |
| 1 | 224 | cows | 19 | 21 | 1.1965 | P | P |
| 1 | 224 | cows | 26 | 28 | 0.732 | P | P |
| 1 | 224 | cows | 33 | 35 | 0.3695 | P | P |
| 1 | 224 | cows | 40 | 42 | 0.098 | P | O |
| 1 | 224 | cows | 49 | 49 | 0.067 | P | O |
| 1 | 224 | cows | 58 | 63 | 0.0435 | O | O |
| 1 | 224 | cows | 68 | 70 | 0.0305 | O | O |
| 1 | 227 | cows | 3 | 7 | 3.7015 | P | P |
| 1 | 227 | cows | 11 | 14 | 3.2755 | P | P |
| 1 | 227 | cows | 17 | 21 | 2.08 | P | P |
| 1 | 227 | cows | 24 | 28 | 1.32 | P | P |
| 1 | 227 | cows | 31 | 35 | 0.4785 | P | P |
| 1 | 227 | cows | 38 | 42 | 0.422 | P | O |
| 1 | 227 | cows | 45 | 49 | 0.236 | P | O |
| 1 | 227 | cows | 52 | 56 | 0.0775 | P | O |
| 1 | 227 | cows | 59 | 63 | 0.0525 | O | O |
| 1 | 227 | cows | 66 | 70 | 0.018 | O | O |
| 1 | 227 | cows | 75 | 77 | 0.0225 | O | O |
| 1 | 228 | cows | 2 | 7 | 3.702 | P | P |
| 1 | 228 | cows | 10 | 14 | 3.4015 | P | P |
| 1 | 228 | cows | 16 | 21 | 1.324 | P | P |
| 1 | 228 | cows | 23 | 28 | 1.7835 | P | P |
| 1 | 228 | cows | 30 | 35 | 0.577 | P | P |
| 1 | 228 | cows | 37 | 42 | 0.518 | P | O |
| 1 | 228 | cows | 44 | 49 | 0.1695 | P | O |
| 1 | 228 | cows | 51 | 56 | 0.0915 | P | O |
| 1 | 228 | cows | 58 | 63 | 0.0605 | P | O |
| 1 | 228 | cows | 65 | 70 | 0.033 | P | O |
| 1 | 228 | cows | 74 | 77 | 0.032 | O | O |
| 1 | 248 | cows | 20 | 21 | 2.7855 | P | P |
| 1 | 248 | cows | 28 | 28 | 1.701 | P | P |
| 1 | 248 | cows | 34 | 35 | 2.7395 | P | P |
| 1 | 248 | cows | 41 | 42 | 0.316 | P | P |
| 1 | 248 | cows | 48 | 49 | 0.1325 | P | O |
| 1 | 248 | cows | 55 | 56 | 0.1445 | P | O |
| 1 | 248 | cows | 62 | 63 | 0.078 | P | O |
| 1 | 248 | cows | 69 | 70 | 0.041 | P | O |
| 1 | 248 | cows | 76 | 77 | 0.0245 | P | O |
| 1 | 248 | cows | 83 | 84 | 0.019 | P | O |
| 1 | 248 | cows | 92 | 84 | 0.0195 | O | O |
| 1 | 248 | cows | 101 | 84 | 0.0215 | O | O |
| 1 | 248 | cows | 111 | 84 | 0.019 | O | O |
| 1 | 249 | cows | 6 | 7 | 3.6565 | P | P |
| 1 | 249 | cows | 12 | 14 | 3.2575 | P | P |
| 1 | 249 | cows | 19 | 21 | 2.5655 | P | P |
| 1 | 249 | cows | 26 | 28 | 1.665 | P | P |
| 1 | 249 | cows | 33 | 35 | 1.4355 | P | P |
| 1 | 249 | cows | 40 | 42 | 0.805 | P | P |
| 1 | 249 | cows | 47 | 49 | 0.361 | P | P |
| 1 | 249 | cows | 54 | 56 | 0.2275 | P | O |
| 1 | 249 | cows | 61 | 63 | 0.059 | P | O |
| 1 | 249 | cows | 70 | 70 | 0.051 | P | O |
| 1 | 266 | cows | 0 | 7 | 3.712 | P | P |
| 1 | 266 | cows | 7 | 7 | 3.403 | P | P |
| 1 | 266 | cows | 14 | 14 | 2.8405 | P | P |
| 1 | 266 | cows | 21 | 21 | 1.234 | P | P |
| 1 | 266 | cows | 28 | 28 | 0.6005 | P | P |
| 1 | 266 | cows | 35 | 35 | 0.244 | P | O |
| 1 | 266 | cows | 44 | 49 | 0.142 | P | O |
| 1 | 266 | cows | 56 | 56 | 0.0555 | P | O |
| 1 | 266 | cows | 63 | 63 | 0.0375 | P | O |
| 1 | 289 | cows | 2 | 7 | 3.488 | P | P |
| 1 | 289 | cows | 10 | 14 | 3.3195 | P | P |
| 1 | 289 | cows | 16 | 21 | 2.102 | P | P |
| 1 | 289 | cows | 23 | 28 | 1.9295 | P | P |
| 1 | 289 | cows | 30 | 35 | 0.748 | P | P |
| 1 | 289 | cows | 37 | 42 | 0.6325 | P | O |
| 1 | 289 | cows | 44 | 49 | 0.182 | P | O |
| 1 | 289 | cows | 51 | 56 | 0.091 | P | O |
| 1 | 289 | cows | 58 | 63 | 0.055 | O | O |
| 1 | 289 | cows | 65 | 70 | 0.028 | O | O |
| 1 | 289 | cows | 74 | 77 | 0.02 | O | O |
| 1 | 307 | cows | 6 | 7 | 3.4345 | P | P |
| 1 | 307 | cows | 14 | 14 | 2.41 | P | P |
| 1 | 307 | cows | 20 | 21 | 1.241 | P | P |
| 1 | 307 | cows | 27 | 28 | 0.414 | P | P |
| 1 | 307 | cows | 34 | 35 | 0.254 | P | P |
| 1 | 307 | cows | 41 | 42 | 0.213 | P | O |
| 1 | 307 | cows | 48 | 49 | 0.1245 | P | O |
| 1 | 307 | cows | 55 | 56 | 0.0415 | P | O |
| 1 | 307 | cows | 62 | 63 | 0.0305 | P | O |
| 1 | 307 | cows | 69 | 70 | 0.019 | O | O |
| 1 | 307 | cows | 78 | 84 | 0.016 | O | O |
| 1 | 330 | cows | 2 | 7 | 3.788 | P | P |
| 1 | 330 | cows | 10 | 14 | 3.428 | P | P |
| 1 | 330 | cows | 16 | 21 | 2.8585 | P | P |
| 1 | 330 | cows | 23 | 28 | 1.4985 | P | P |
| 1 | 330 | cows | 30 | 35 | 0.7305 | P | P |
| 1 | 330 | cows | 37 | 42 | 0.5365 | P | O |
| 1 | 330 | cows | 44 | 49 | 0.2625 | P | O |
| 1 | 330 | cows | 51 | 56 | 0.09 | P | O |
| 1 | 330 | cows | 58 | 63 | 0.0505 | P | P |
| 1 | 330 | cows | 65 | 70 | 0.032 | P | O |
| 1 | 330 | cows | 74 | 77 | 0.023 | O | O |
| 1 | 362 | cows | 2 | 7 | 3.7855 | P | P |
| 1 | 362 | cows | 10 | 14 | 2.54 | P | P |
| 1 | 362 | cows | 16 | 21 | 3.233 | P | P |
| 1 | 362 | cows | 23 | 28 | 0.7165 | P | O |
| 1 | 362 | cows | 30 | 35 | 0.2905 | P | O |
| 1 | 362 | cows | 37 | 42 | 0.217 | P | O |
| 1 | 362 | cows | 44 | 49 | 0.112 | P | O |
| 1 | 362 | cows | 51 | 56 | 0.0275 | P | O |
| 1 | 362 | cows | 58 | 63 | 0.026 | O | O |
| 1 | 362 | cows | 65 | 70 | 0.016 | O | O |
| 1 | 362 | cows | 74 | 77 | 0.0155 | O | O |
| 1 | 385 | cows | 2 | 7 | 3.7535 | P | P |
| 1 | 385 | cows | 9 | 14 | 2.837 | P | P |
| 1 | 385 | cows | 16 | 21 | 1.1285 | P | P |
| 1 | 385 | cows | 30 | 35 | 0.416 | P | P |
| 1 | 385 | cows | 39 | 42 | 0.1765 | P | O |
| 1 | 385 | cows | 48 | 49 | 0.028 | P | O |
| 1 | 385 | cows | 58 | 63 | 0.055 | P | O |
| 1 | 385 | cows | . | . | . | P | P |
| 1 | 387 | cows | 2 | 7 | 3.7155 | P | P |
| 1 | 387 | cows | 10 | 14 | 1.7595 | P | P |
| 1 | 387 | cows | 16 | 21 | 2.4715 | P | P |
| 1 | 387 | cows | 23 | 28 | 0.315 | P | O |
| 1 | 387 | cows | 30 | 35 | 0.1925 | P | O |
| 1 | 387 | cows | 37 | 42 | 0.271 | P | O |
| 1 | 387 | cows | 44 | 49 | 0.1745 | P | O |
| 1 | 387 | cows | 51 | 56 | 0.08 | P | O |
| 1 | 387 | cows | 65 | 70 | 0.047 | O | O |
| 1 | 387 | cows | 74 | 77 | 0.089 | O | O |
| 1 | 387 | cows | . | . | . | P | O |
| 1 | 390 | cows | 7 | 7 | 3.4335 | P | P |
| 1 | 390 | cows | 14 | 14 | 2.264 | P | P |
| 1 | 390 | cows | 21 | 21 | 1.019 | O | P |
| 1 | 390 | cows | 30 | 35 | 0.667 | P | P |
| 1 | 390 | cows | 42 | 42 | 0.1975 | P | P |
| 1 | 390 | cows | 49 | 49 | 0.106 | P | P |
| 1 | 401 | cows | 18 | 21 | 1.205 | P | P |
| 1 | 401 | cows | 25 | 28 | 0.6815 | P | P |
| 1 | 401 | cows | 32 | 35 | 0.227 | P | O |
| 1 | 401 | cows | 39 | 42 | 0.1165 | P | O |
| 1 | 401 | cows | 46 | 49 | 0.067 | P | O |
| 1 | 401 | cows | 53 | 56 | 0.0525 | P | O |
| 1 | 401 | cows | 60 | 63 | 0.028 | O | O |
| 1 | 401 | cows | 69 | 70 | 0.073 | O | O |
| 1 | 401 | cows | 78 | 84 | 0.0265 | O | O |
| 1 | 401 | cows | 88 | 84 | 0.02 | O | O |
| 1 | 403 | cows | 3 | 7 | 3.708 | P | P |
| 1 | 403 | cows | 11 | 14 | 2.415 | P | P |
| 1 | 403 | cows | 17 | 21 | 2.4115 | P | P |
| 1 | 403 | cows | 24 | 28 | 0.6855 | P | P |
| 1 | 403 | cows | 31 | 35 | 0.342 | P | O |
| 1 | 403 | cows | 38 | 42 | 0.1155 | P | O |
| 1 | 403 | cows | 45 | 49 | 0.149 | O | O |
| 1 | 403 | cows | 52 | 56 | 0.0515 | O | O |
| 1 | 403 | cows | 59 | 63 | 0.0355 | O | O |
| 1 | 403 | cows | 66 | 70 | 0.023 | O | O |
| 1 | 403 | cows | 75 | 77 | 0.019 | O | O |
| 1 | 403 | cows | 84 | 84 | 0.021 | O | O |
| 1 | 403 | cows | 94 | 84 | 0.022 | O | O |
| 1 | 404 | cows | 7 | 7 | 3.51 | P | P |
| 1 | 404 | cows | 15 | 21 | 1.78 | P | P |
| 1 | 404 | cows | 28 | 28 | 0.744 | P | P |
| 1 | 404 | cows | 35 | 35 | 0.3125 | P | O |
| 1 | 404 | cows | 42 | 42 | 0.1655 | P | O |
| 1 | 404 | cows | 49 | 49 | 0.1795 | P | O |
| 1 | 404 | cows | 56 | 56 | 0.066 | P | O |
| 1 | 404 | cows | 63 | 63 | 0.046 | P | O |
| 1 | 404 | cows | 70 | 70 | 0.025 | O | O |
| 1 | 404 | cows | 79 | 84 | 0.0175 | . | . |
| 1 | 408 | cows | 49 | 49 | . | O | . |
| 1 | 408 | cows | 84 | 84 | . | O | O |
| 1 | 408 | cows | 94 | 84 | . | O | O |
| 1 | 415 | cows | 2 | 7 | 3.5575 | P | P |
| 1 | 415 | cows | 10 | 14 | 2.0355 | P | P |
| 1 | 415 | cows | 16 | 21 | 0.964 | P | P |
| 1 | 415 | cows | 23 | 28 | 0.373 | P | O |
| 1 | 415 | cows | 30 | 35 | 0.157 | P | O |
| 1 | 415 | cows | 37 | 42 | 0.0735 | O | O |
| 1 | 415 | cows | 44 | 49 | 0.036 | O | O |
| 1 | 415 | cows | 51 | 56 | 0.0235 | O | O |
| 1 | 415 | cows | 58 | 63 | 0.02 | O | O |
| 1 | 415 | cows | 65 | 70 | 0.019 | O | O |
| 1 | 415 | cows | 74 | 77 | 0.026 | O | O |
| 1 | 416 | cows | 5 | 7 | 3.7605 | P | P |
| 1 | 416 | cows | 12 | 14 | 3.239 | P | P |
| 1 | 416 | cows | 19 | 21 | 2.295 | P | P |
| 1 | 416 | cows | 26 | 28 | 1.281 | P | P |
| 1 | 416 | cows | 33 | 35 | 0.878 | P | P |
| 1 | 416 | cows | 42 | 42 | 0.315 | P | P |
| 1 | 416 | cows | 51 | 56 | 0.203 | P | O |
| 1 | 416 | cows | 61 | 63 | 0.0875 | P | P |
| 1 | 424 | cows | 5 | 7 | 3.433 | P | P |
| 1 | 424 | cows | 12 | 14 | 2.4355 | P | P |
| 1 | 424 | cows | 19 | 21 | 1.4885 | P | P |
| 1 | 424 | cows | 26 | 28 | 0.747 | P | P |
| 1 | 424 | cows | 33 | 35 | 0.307 | P | O |
| 1 | 424 | cows | 42 | 42 | 0.216 | P | O |
| 1 | 424 | cows | 51 | 56 | 0.079 | P | O |
| 1 | 424 | cows | 61 | 63 | 0.0405 | O | O |
| 1 | 437 | cows | 3 | 7 | 3.72 | P | P |
| 1 | 437 | cows | 11 | 14 | 3.2815 | P | P |
| 1 | 437 | cows | 17 | 21 | 2.2695 | P | P |
| 1 | 437 | cows | 24 | 28 | 1.3125 | P | P |
| 1 | 437 | cows | 31 | 35 | 0.604 | P | P |
| 1 | 437 | cows | 38 | 42 | 0.344 | P | O |
| 1 | 437 | cows | 45 | 49 | 0.309 | P | O |
| 1 | 437 | cows | 52 | 56 | 0.073 | P | O |
| 1 | 437 | cows | 59 | 63 | 0.039 | P | O |
| 1 | 437 | cows | 66 | 70 | 0.0225 | P | O |
| 1 | 437 | cows | 75 | 77 | 0.0215 | O | O |
| 1 | 439 | cows | 9 | 14 | 3.3985 | P | P |
| 1 | 439 | cows | 15 | 21 | 2.702 | P | P |
| 1 | 439 | cows | 22 | 28 | 1.413 | P | P |
| 1 | 439 | cows | 29 | 35 | 0.483 | P | P |
| 1 | 439 | cows | 36 | 42 | 0.423 | P | O |
| 1 | 439 | cows | 43 | 49 | 0.3045 | P | O |
| 1 | 439 | cows | 50 | 56 | 0.1025 | P | O |
| 1 | 439 | cows | 57 | 63 | 0.0575 | P | O |
| 1 | 439 | cows | 64 | 70 | 0.031 | P | O |
| 1 | 439 | cows | 73 | 77 | 0.0285 | P | O |
| 1 | 439 | cows | 82 | 84 | 0.02 | O | O |
| 1 | 439 | cows | 92 | 84 | 0.0155 | O | O |
| 1 | 480 | cows | 6 | 7 | 3.774 | P | P |
| 1 | 480 | cows | 14 | 14 | 3.5545 | P | P |
| 1 | 480 | cows | 20 | 21 | 3.189 | P | P |
| 1 | 480 | cows | 27 | 28 | 2.0845 | P | P |
| 1 | 480 | cows | 34 | 35 | 1.056 | P | P |
| 1 | 480 | cows | 41 | 42 | 0.6335 | P | O |
| 1 | 480 | cows | 48 | 49 | 0.5175 | P | O |
| 1 | 480 | cows | 55 | 56 | 0.1755 | P | O |
| 1 | 480 | cows | 62 | 63 | 0.0725 | P | O |
| 1 | 480 | cows | 69 | 70 | 0.04 | O | O |
| 1 | 480 | cows | 78 | 84 | 0.0405 | O | O |
| 1 | 502 | cows | 5 | 7 | 3.441 | P | P |
| 1 | 502 | cows | 12 | 14 | 3.0355 | P | P |
| 1 | 502 | cows | 19 | 21 | 2.126 | P | P |
| 1 | 502 | cows | 26 | 28 | 1.364 | P | P |
| 1 | 502 | cows | 33 | 35 | 0.505 | P | P |
| 1 | 502 | cows | 42 | 42 | 0.416 | P | P |
| 1 | 502 | cows | 54 | 56 | 0.1505 | O | O |
| 1 | 502 | cows | 61 | 63 | 0.105 | P | O |
| 1 | 512 | cows | 9 | 14 | 3.55 | P | P |
| 1 | 512 | cows | 15 | 21 | 2.263 | P | P |
| 1 | 512 | cows | 22 | 28 | 1.3845 | P | P |
| 1 | 512 | cows | 29 | 35 | 0.698 | P | P |
| 1 | 512 | cows | 36 | 42 | 0.3475 | P | O |
| 1 | 512 | cows | 43 | 49 | 0.271 | P | O |
| 1 | 512 | cows | 50 | 56 | 0.0935 | P | O |
| 1 | 512 | cows | 57 | 63 | 0.0445 | P | O |
| 1 | 512 | cows | 64 | 70 | 0.024 | O | O |
| 1 | 512 | cows | 73 | 77 | 0.0245 | O | O |
| 1 | 517 | cows | 6 | 7 | 3.778 | P | P |
| 1 | 517 | cows | 12 | 14 | 2.1375 | P | P |
| 1 | 517 | cows | 19 | 21 | 2.8905 | P | P |
| 1 | 517 | cows | 26 | 28 | 2.075 | P | P |
| 1 | 517 | cows | 33 | 35 | 0.9925 | P | P |
| 1 | 517 | cows | 40 | 42 | 0.9065 | P | P |
| 1 | 517 | cows | 47 | 49 | 0.3015 | P | P |
| 1 | 517 | cows | 54 | 56 | 0.197 | P | O |
| 1 | 517 | cows | 61 | 63 | 0.085 | P | O |
| 1 | 517 | cows | 70 | 70 | 0.0715 | O | O |
| 1 | 532 | cows | 4 | 7 | 3.4795 | P | P |
| 1 | 532 | cows | 11 | 14 | 3.3595 | P | P |
| 1 | 532 | cows | 18 | 21 | 1.623 | P | P |
| 1 | 532 | cows | 27 | 28 | 1.21 | P | P |
| 1 | 532 | cows | 39 | 42 | 0.415 | P | P |
| 1 | 532 | cows | 46 | 49 | 0.194 | P | O |
| 1 | 537 | cows | 9 | 14 | 3.068 | P | P |
| 1 | 537 | cows | 15 | 21 | 1.34 | P | P |
| 1 | 537 | cows | 22 | 28 | 1.4845 | P | P |
| 1 | 537 | cows | 29 | 35 | 0.634 | P | P |
| 1 | 537 | cows | 36 | 42 | 0.344 | P | P |
| 1 | 537 | cows | 43 | 49 | 0.358 | P | O |
| 1 | 537 | cows | 50 | 56 | 0.0915 | P | O |
| 1 | 537 | cows | 57 | 63 | 0.0545 | P | O |
| 1 | 537 | cows | 64 | 70 | 0.0305 | P | O |
| 1 | 537 | cows | 73 | 77 | 0.0435 | P | O |
| 1 | 542 | cows | 7 | 7 | 0.025 | . | . |
| 1 | 542 | cows | 11 | 14 | 1.6985 | P | P |
| 1 | 542 | cows | 18 | 21 | 1.4335 | P | P |
| 1 | 542 | cows | 25 | 28 | 0.4605 | P | P |
| 1 | 542 | cows | 32 | 35 | 0.3035 | P | P |
| 1 | 542 | cows | 39 | 42 | 0.15 | P | P |
| 1 | 542 | cows | 46 | 49 | 0.0765 | P | O |
| 1 | 542 | cows | 53 | 56 | 0.0595 | O | O |
| 1 | 542 | cows | 60 | 63 | 0.0315 | O | O |
| 1 | 542 | cows | 69 | 70 | 0.028 | O | O |
| 1 | 543 | cows | 3 | 7 | 3.811 | P | P |
| 1 | 543 | cows | 11 | 14 | 3.161 | P | P |
| 1 | 543 | cows | 17 | 21 | 0.8505 | P | P |
| 1 | 543 | cows | 24 | 28 | 1.176 | P | P |
| 1 | 543 | cows | 31 | 35 | 0.3535 | P | P |
| 1 | 543 | cows | 38 | 42 | 0.2145 | P | O |
| 1 | 543 | cows | 45 | 49 | 0.2025 | P | O |
| 1 | 543 | cows | 52 | 56 | 0.0675 | P | O |
| 1 | 543 | cows | 59 | 63 | 0.036 | P | O |
| 1 | 543 | cows | 66 | 70 | 0.025 | P | O |
| 1 | 543 | cows | 75 | 77 | 0.0335 | O | O |
| 1 | 546 | cows | 8 | 14 | 3.637 | P | P |
| 1 | 546 | cows | 16 | 21 | 2.132 | P | P |
| 1 | 546 | cows | 22 | 28 | 1.4175 | P | P |
| 1 | 546 | cows | 29 | 35 | 0.721 | P | O |
| 1 | 546 | cows | 36 | 42 | 0.265 | P | P |
| 1 | 546 | cows | 43 | 49 | 0.149 | P | O |
| 1 | 546 | cows | 50 | 56 | 0.141 | P | O |
| 1 | 546 | cows | 57 | 63 | 0.051 | O | O |
| 1 | 546 | cows | 64 | 70 | 0.0285 | O | O |
| 1 | 546 | cows | 71 | 77 | 0.019 | O | O |
| 1 | 546 | cows | . | . | . | P | . |
| 1 | 560 | cows | 11 | 14 | 3.3645 | P | P |
| 1 | 560 | cows | 19 | 21 | 2.3785 | P | P |
| 1 | 560 | cows | 25 | 28 | 1.3225 | P | P |
| 1 | 560 | cows | 32 | 35 | 0.7035 | P | O |
| 1 | 560 | cows | 39 | 42 | 0.336 | P | O |
| 1 | 560 | cows | 46 | 49 | 0.142 | P | O |
| 1 | 560 | cows | 53 | 56 | 0.1775 | P | O |
| 1 | 560 | cows | 60 | 63 | 0.058 | P | O |
| 1 | 560 | cows | 67 | 70 | 0.033 | O | O |
| 1 | 560 | cows | 74 | 77 | 0.023 | O | O |
| 1 | 564 | cows | 4 | 7 | 3.318 | P | P |
| 1 | 564 | cows | 11 | 14 | 3.1615 | P | P |
| 1 | 564 | cows | 18 | 21 | 1.36 | P | P |
| 1 | 564 | cows | 27 | 28 | 1.4295 | . | P |
| 1 | 564 | cows | 39 | 42 | 0.387 | P | O |
| 1 | 564 | cows | 46 | 49 | 0.165 | P | P |
| 1 | 599 | cows | 6 | 7 | 3.583 | P | P |
| 1 | 599 | cows | 14 | 14 | 2.65 | P | P |
| 1 | 599 | cows | 20 | 21 | 1.9235 | P | P |
| 1 | 599 | cows | 27 | 28 | 0.864 | P | P |
| 1 | 599 | cows | 34 | 35 | 0.279 | P | P |
| 1 | 599 | cows | 41 | 42 | 0.1525 | P | O |
| 1 | 599 | cows | 48 | 49 | 0.233 | P | O |
| 1 | 599 | cows | 55 | 56 | 0.054 | P | O |
| 1 | 599 | cows | 62 | 63 | 0.031 | O | O |
| 1 | 599 | cows | 69 | 70 | 0.02 | O | O |
| 1 | 599 | cows | 78 | 84 | 0.0245 | O | O |
| 1 | 607 | cows | 9 | 14 | 3.4575 | P | P |
| 1 | 607 | cows | 15 | 21 | 2.448 | P | P |
| 1 | 607 | cows | 22 | 28 | 1.478 | P | P |
| 1 | 607 | cows | 29 | 35 | 0.6795 | P | P |
| 1 | 607 | cows | 36 | 42 | 0.511 | P | O |
| 1 | 607 | cows | 43 | 49 | 0.271 | P | O |
| 1 | 607 | cows | 50 | 56 | 0.1005 | P | P |
| 1 | 607 | cows | 57 | 63 | 0.0635 | P | O |
| 1 | 607 | cows | 64 | 70 | 0.0265 | P | O |
| 1 | 607 | cows | 73 | 77 | 0.0265 | O | O |
| 1 | 626 | cows | 4 | 7 | 3.3765 | P | P |
| 1 | 626 | cows | 11 | 14 | 3.132 | P | P |
| 1 | 626 | cows | 18 | 21 | 1.5195 | P | P |
| 1 | 626 | cows | 27 | 28 | 1.1435 | P | P |
| 1 | 626 | cows | 39 | 42 | 0.362 | P | O |
| 1 | 626 | cows | 46 | 49 | 0.173 | P | P |
| 1 | 638 | cows | 7 | 7 | 3.5595 | P | P |
| 1 | 638 | cows | 15 | 21 | 3.062 | P | P |
| 1 | 638 | cows | 21 | 21 | 2.334 | P | P |
| 1 | 638 | cows | 28 | 28 | 0.7775 | P | P |
| 1 | 638 | cows | 35 | 35 | 0.329 | P | P |
| 1 | 638 | cows | 42 | 42 | 0.21 | P | O |
| 1 | 638 | cows | 49 | 49 | 0.132 | P | O |
| 1 | 638 | cows | 56 | 56 | 0.0565 | P | O |
| 1 | 638 | cows | 63 | 63 | 0.0255 | P | O |
| 1 | 638 | cows | 70 | 70 | 0.02 | 0 | O |
| 1 | 646 | cows | 2 | 7 | 3.783 | P | P |
| 1 | 646 | cows | 9 | 14 | 1.899 | P | P |
| 1 | 646 | cows | 16 | 21 | 1.8595 | P | P |
| 1 | 646 | cows | 23 | 28 | 1.0425 | P | P |
| 1 | 646 | cows | 30 | 35 | 0.4 | P | P |
| 1 | 646 | cows | 39 | 42 | 0.2705 | P | O |
| 1 | 646 | cows | 51 | 56 | 0.0965 | O | O |
| 1 | 646 | cows | 58 | 63 | 0.044 | O | O |
| 1 | 651 | cows | 7 | 7 | 3.424 | P | P |
| 1 | 651 | cows | 13 | 14 | 1.393 | P | P |
| 1 | 651 | cows | 20 | 21 | 1.3075 | P | P |
| 1 | 651 | cows | 27 | 28 | 0.5715 | P | P |
| 1 | 651 | cows | 34 | 35 | 0.2715 | P | O |
| 1 | 651 | cows | 41 | 42 | 0.124 | P | P |
| 1 | 651 | cows | 48 | 49 | 0.079 | P | O |
| 1 | 651 | cows | 55 | 56 | 0.042 | P | O |
| 1 | 651 | cows | 62 | 63 | 0.029 | P | O |
| 1 | 651 | cows | 71 | 77 | 0.025 | P | O |
| 1 | 659 | cows | 5 | 7 | 3.5665 | P | P |
| 1 | 659 | cows | 11 | 14 | 1.71 | P | P |
| 1 | 659 | cows | 18 | 21 | 1.603 | P | P |
| 1 | 659 | cows | 25 | 28 | 0.8145 | P | P |
| 1 | 659 | cows | 32 | 35 | 0.33 | P | O |
| 1 | 659 | cows | 39 | 42 | 0.242 | P | P |
| 1 | 659 | cows | 46 | 49 | 0.0885 | P | O |
| 1 | 659 | cows | 53 | 56 | 0.06 | P | O |
| 1 | 659 | cows | 60 | 63 | 0.0305 | P | O |
| 1 | 659 | cows | 69 | 70 | 0.0255 | O | O |
| 1 | 665 | cows | 7 | 7 | 1.4675 | P | P |
| 1 | 665 | cows | 14 | 14 | 2.0925 | P | P |
| 1 | 665 | cows | 21 | 21 | 1.363 | P | P |
| 1 | 665 | cows | 28 | 28 | 0.814 | P | P |
| 1 | 665 | cows | 35 | 35 | 0.3405 | P | O |
| 1 | 665 | cows | 42 | 42 | 0.118 | P | O |
| 1 | 665 | cows | 49 | 49 | 0.0755 | P | O |
| 1 | 665 | cows | 56 | 56 | 0.0785 | P | O |
| 1 | 665 | cows | 65 | 70 | 0.0595 | P | O |
| 1 | 672 | cows | 6 | 7 | 3.344 | P | P |
| 1 | 672 | cows | 13 | 14 | 1.8215 | P | P |
| 1 | 672 | cows | 20 | 21 | 1.243 | P | P |
| 1 | 672 | cows | 27 | 28 | 1.011 | P | P |
| 1 | 672 | cows | 36 | 42 | 0.309 | P | O |
| 1 | 672 | cows | 48 | 49 | 0.0965 | P | P |
| 1 | 672 | cows | 55 | 56 | 0.0495 | P | O |
| 1 | 673 | cows | 7 | 7 | 3.297 | P | P |
| 1 | 673 | cows | 14 | 14 | 3.064 | P | P |
| 1 | 673 | cows | 21 | 21 | 2.3315 | P | P |
| 1 | 673 | cows | 30 | 35 | 0.7605 | P | P |
| 1 | 673 | cows | 42 | 42 | 0.2955 | P | P |
| 1 | 673 | cows | 49 | 49 | 0.1525 | P | P |
| 1 | 683 | cows | 8 | 14 | 3.381 | P | P |
| 1 | 683 | cows | 14 | 14 | 2.1345 | P | P |
| 1 | 683 | cows | 21 | 21 | 0.8 | P | P |
| 1 | 683 | cows | 28 | 28 | 0.6625 | P | P |
| 1 | 683 | cows | 35 | 35 | 0.2045 | P | O |
| 1 | 683 | cows | 42 | 42 | 0.1455 | P | O |
| 1 | 683 | cows | 49 | 49 | 0.052 | O | O |
| 1 | 683 | cows | 56 | 56 | 0.0445 | O | O |
| 1 | 683 | cows | 63 | 63 | 0.041 | O | O |
| 1 | 683 | cows | 72 | 77 | 0.032 | O | O |
| 1 | 690 | cows | 4 | 7 | 3.6355 | P | P |
| 1 | 690 | cows | 12 | 14 | 3.1755 | P | P |
| 1 | 690 | cows | 18 | 21 | 1.7375 | P | P |
| 1 | 690 | cows | 25 | 28 | 0.919 | P | P |
| 1 | 690 | cows | 32 | 35 | 0.411 | P | P |
| 1 | 690 | cows | 39 | 42 | 0.215 | P | O |
| 1 | 690 | cows | 46 | 49 | 0.134 | P | O |
| 1 | 690 | cows | 53 | 56 | 0.0515 | P | O |
| 1 | 690 | cows | 60 | 63 | 0.036 | O | O |
| 1 | 690 | cows | 67 | 70 | 0.03 | O | O |
| 1 | 690 | cows | 76 | 77 | 0.0195 | O | O |
| 1 | 697 | cows | 6 | 7 | 3.6205 | P | P |
| 1 | 697 | cows | 12 | 14 | 3.118 | P | P |
| 1 | 697 | cows | 19 | 21 | 1.759 | P | P |
| 1 | 697 | cows | 26 | 28 | 1.1905 | P | P |
| 1 | 697 | cows | 33 | 35 | 0.941 | P | P |
| 1 | 697 | cows | 40 | 42 | 0.3395 | P | O |
| 1 | 697 | cows | 47 | 49 | 0.12 | P | O |
| 1 | 697 | cows | 54 | 56 | 0.06 | P | O |
| 1 | 697 | cows | 61 | 63 | 0.055 | O | O |
| 1 | 697 | cows | 70 | 70 | 0.036 | P | O |
| 1 | 717 | cows | 3 | 7 | 3.569 | P | P |
| 1 | 717 | cows | 11 | 14 | 2.767 | P | P |
| 1 | 717 | cows | 17 | 21 | 2.3425 | P | P |
| 1 | 717 | cows | 24 | 28 | 0.472 | P | O |
| 1 | 717 | cows | 31 | 35 | 0.2805 | P | P |
| 1 | 717 | cows | 38 | 42 | 0.116 | P | O |
| 1 | 717 | cows | 45 | 49 | 0.068 | P | O |
| 1 | 717 | cows | 52 | 56 | 0.026 | P | O |
| 1 | 717 | cows | 59 | 63 | 0.023 | O | O |
| 1 | 717 | cows | 66 | 70 | 0.0195 | O | O |
| 1 | 717 | cows | 75 | 77 | 0.02 | O | O |
| 1 | 722 | cows | 6 | 7 | 3.4925 | P | P |
| 1 | 722 | cows | 12 | 14 | 2.4755 | P | P |
| 1 | 722 | cows | 19 | 21 | 1.533 | P | P |
| 1 | 722 | cows | 26 | 28 | 1.015 | P | P |
| 1 | 722 | cows | 33 | 35 | 0.6675 | P | O |
| 1 | 722 | cows | 40 | 42 | 0.262 | P | P |
| 1 | 722 | cows | 47 | 49 | 0.0845 | O | O |
| 1 | 722 | cows | 54 | 56 | 0.0495 | O | O |
| 1 | 722 | cows | 61 | 63 | 0.047 | O | O |
| 1 | 722 | cows | 70 | 70 | 0.0285 | O | O |
| 1 | 725 | cows | 7 | 7 | 3.8555 | P | P |
| 1 | 725 | cows | 14 | 14 | 2.4365 | P | P |
| 1 | 725 | cows | 21 | 21 | 1.9405 | P | P |
| 1 | 725 | cows | 28 | 28 | 1.347 | P | P |
| 1 | 725 | cows | 37 | 42 | 0.502 | P | O |
| 1 | 725 | cows | 49 | 49 | 0.1375 | P | O |
| 1 | 725 | cows | 56 | 56 | 0.0805 | P | O |
| 1 | 738 | cows | 4 | 7 | 3.8005 | P | P |
| 1 | 738 | cows | 12 | 14 | 3.201 | P | P |
| 1 | 738 | cows | 18 | 21 | 1.7635 | P | P |
| 1 | 738 | cows | 25 | 28 | 1.202 | P | P |
| 1 | 738 | cows | 32 | 35 | 0.5975 | P | P |
| 1 | 738 | cows | 39 | 42 | 0.308 | P | O |
| 1 | 738 | cows | 46 | 49 | 0.1825 | P | P |
| 1 | 738 | cows | 53 | 56 | 0.0955 | P | P |
| 1 | 738 | cows | 60 | 63 | 0.06 | P | O |
| 1 | 738 | cows | 67 | 70 | 0.055 | O | O |
| 1 | 738 | cows | 76 | 77 | 0.0335 | O | O |
| 1 | 752 | cows | 6 | 7 | 3.4315 | P | P |
| 1 | 752 | cows | 14 | 14 | 2.2395 | P | P |
| 1 | 752 | cows | 20 | 21 | 2.3745 | P | P |
| 1 | 752 | cows | 27 | 28 | 0.504 | P | P |
| 1 | 752 | cows | 34 | 35 | 0.277 | P | P |
| 1 | 752 | cows | 41 | 42 | 0.2865 | P | O |
| 1 | 752 | cows | 48 | 49 | 0.0885 | P | O |
| 1 | 752 | cows | 55 | 56 | 0.036 | P | O |
| 1 | 752 | cows | 62 | 63 | 0.0275 | O | O |
| 1 | 752 | cows | 69 | 70 | 0.021 | O | O |
| 1 | 752 | cows | 78 | 84 | 0.0195 | O | O |
| 1 | 769 | cows | 1 | 7 | 3.8385 | P | P |
| 1 | 769 | cows | 8 | 14 | 3.349 | P | P |
| 1 | 769 | cows | 15 | 21 | 2.2485 | P | P |
| 1 | 769 | cows | 22 | 28 | 1.985 | P | P |
| 1 | 769 | cows | 29 | 35 | 1.3945 | P | P |
| 1 | 769 | cows | 38 | 42 | 0.6305 | P | P |
| 1 | 769 | cows | 50 | 56 | 0.181 | P | O |
| 1 | 769 | cows | 57 | 63 | 0.1185 | P | O |
| 1 | 770 | cows | 2 | 7 | 3.5985 | P | P |
| 1 | 770 | cows | 9 | 14 | 2.738 | P | P |
| 1 | 770 | cows | 16 | 21 | 1.1965 | P | P |
| 1 | 770 | cows | 23 | 28 | 1.0185 | P | P |
| 1 | 770 | cows | 30 | 35 | 0.554 | P | O |
| 1 | 770 | cows | 39 | 42 | 0.2085 | P | O |
| 1 | 770 | cows | 51 | 56 | 0.066 | P | O |
| 1 | 770 | cows | 58 | 63 | 0.037 | O | O |
| 1 | 772 | cows | 3 | 7 | 3.801 | P | P |
| 1 | 772 | cows | 11 | 14 | 3.497 | P | P |
| 1 | 772 | cows | 17 | 21 | 2.8355 | P | P |
| 1 | 772 | cows | 24 | 28 | 1.5335 | P | P |
| 1 | 772 | cows | 31 | 35 | 0.759 | P | P |
| 1 | 772 | cows | 38 | 42 | 0.323 | P | O |
| 1 | 772 | cows | 45 | 49 | 0.1675 | P | O |
| 1 | 772 | cows | 52 | 56 | 0.074 | P | O |
| 1 | 772 | cows | 59 | 63 | 0.058 | O | O |
| 1 | 772 | cows | 66 | 70 | 0.0475 | O | O |
| 1 | 772 | cows | 75 | 77 | 0.035 | O | O |
| 1 | 779 | cows | 7 | 7 | 3.605 | P | P |
| 1 | 779 | cows | 13 | 14 | 3.024 | P | P |
| 1 | 779 | cows | 20 | 21 | 1.375 | P | P |
| 1 | 779 | cows | 27 | 28 | 0.6785 | P | P |
| 1 | 779 | cows | 34 | 35 | 0.3755 | P | P |
| 1 | 779 | cows | 41 | 42 | 0.2455 | P | O |
| 1 | 779 | cows | 48 | 49 | 0.059 | P | O |
| 1 | 779 | cows | 55 | 56 | 0.051 | P | O |
| 1 | 779 | cows | 62 | 63 | 0.0315 | O | O |
| 1 | 779 | cows | 71 | 77 | 0.023 | O | O |
| 1 | 787 | cows | 7 | 7 | 3.5095 | P | P |
| 1 | 787 | cows | 14 | 14 | 2.501 | P | P |
| 1 | 787 | cows | 21 | 21 | 1.095 | P | P |
| 1 | 787 | cows | 28 | 28 | 0.661 | P | P |
| 1 | 787 | cows | 35 | 35 | 0.3985 | P | O |
| 1 | 787 | cows | 44 | 49 | 0.167 | P | O |
| 1 | 787 | cows | 56 | 56 | 0.0515 | O | O |
| 1 | 787 | cows | 63 | 63 | 0.0315 | O | O |
| 1 | 797 | cows | . | . | . | P | O |
| 2 | 801 | heifers | 7 | 7 | 2.683 | P | P |
| 2 | 801 | heifers | 14 | 14 | 1.3205 | P | P |
| 2 | 801 | heifers | 21 | 21 | 0.4755 | P | P |
| 2 | 801 | heifers | 28 | 28 | 0.2645 | P | O |
| 2 | 801 | heifers | 35 | 35 | 0.107 | P | O |
| 2 | 801 | heifers | 42 | 42 | 0.089 | P | O |
| 2 | 801 | heifers | 49 | 49 | 0.0375 | P | O |
| 2 | 801 | heifers | 58 | 63 | 0.033 | P | O |
| 2 | 801 | heifers | 70 | 70 | 0.0185 | O | O |
| 2 | 801 | heifers | 77 | 77 | 0.0165 | O | O |
| 2 | 804 | heifers | 8 | 14 | 3.282 | P | P |
| 2 | 804 | heifers | 15 | 21 | 1.6575 | P | P |
| 2 | 804 | heifers | 22 | 28 | 0.908 | P | P |
| 2 | 804 | heifers | 29 | 35 | 0.5275 | P | P |
| 2 | 804 | heifers | 36 | 42 | 0.2115 | P | P |
| 2 | 804 | heifers | 43 | 49 | 0.194 | P | O |
| 2 | 804 | heifers | 50 | 56 | 0.0875 | P | O |
| 2 | 804 | heifers | 59 | 63 | 0.043 | P | O |
| 2 | 804 | heifers | 71 | 77 | 0.0265 | O | O |
| 2 | 804 | heifers | 78 | 84 | 0.017 | O | O |
| 2 | 805 | heifers | 7 | 7 | 2.896 | P | P |
| 2 | 805 | heifers | 14 | 14 | 1.221 | P | P |
| 2 | 805 | heifers | 21 | 21 | 1.255 | P | P |
| 2 | 805 | heifers | 28 | 28 | 0.6145 | P | P |
| 2 | 805 | heifers | 37 | 42 | 0.2105 | P | P |
| 2 | 805 | heifers | 49 | 49 | 0.074 | P | O |
| 2 | 805 | heifers | 56 | 56 | 0.0435 | O | O |
| 2 | 806 | heifers | 18 | 21 | 2.879 | P | P |
| 2 | 806 | heifers | 26 | 28 | 1.486 | P | P |
| 2 | 806 | heifers | 32 | 35 | 0.9525 | P | P |
| 2 | 806 | heifers | 39 | 42 | 0.3385 | P | O |
| 2 | 806 | heifers | 46 | 49 | 0.1935 | P | O |
| 2 | 806 | heifers | 53 | 56 | 0.065 | P | O |
| 2 | 806 | heifers | 60 | 63 | 0.0515 | P | O |
| 2 | 806 | heifers | 67 | 70 | 0.022 | O | O |
| 2 | 806 | heifers | 74 | 77 | 0.0315 | O | O |
| 2 | 806 | heifers | 81 | 84 | 0.022 | O | O |
| 2 | 807 | heifers | 4 | 7 | 3.358 | P | P |
| 2 | 807 | heifers | 11 | 14 | 2.836 | P | P |
| 2 | 807 | heifers | 18 | 21 | 2.1945 | P | P |
| 2 | 807 | heifers | 27 | 28 | 0.74 | P | P |
| 2 | 807 | heifers | 39 | 42 | 0.2095 | P | O |
| 2 | 807 | heifers | 46 | 49 | 0.1155 | O | O |
| 2 | 812 | heifers | 13 | 14 | 3.339 | P | P |
| 2 | 812 | heifers | 21 | 21 | 2.0965 | P | P |
| 2 | 812 | heifers | 27 | 28 | 1.227 | P | P |
| 2 | 812 | heifers | 34 | 35 | 0.382 | P | P |
| 2 | 812 | heifers | 41 | 42 | 0.236 | P | O |
| 2 | 812 | heifers | 48 | 49 | 0.0765 | P | O |
| 2 | 812 | heifers | 55 | 56 | 0.065 | O | O |
| 2 | 812 | heifers | 62 | 63 | 0.0215 | O | O |
| 2 | 812 | heifers | 69 | 70 | 0.0295 | O | O |
| 2 | 812 | heifers | 76 | 77 | 0.0225 | O | O |
| 2 | 814 | heifers | 13 | 14 | 2.187 | P | P |
| 2 | 814 | heifers | 21 | 21 | 0.9385 | P | P |
| 2 | 814 | heifers | 27 | 28 | 1.5035 | P | P |
| 2 | 814 | heifers | 34 | 35 | 0.2455 | P | P |
| 2 | 814 | heifers | 41 | 42 | 0.1885 | P | O |
| 2 | 814 | heifers | 48 | 49 | 0.069 | P | O |
| 2 | 814 | heifers | 55 | 56 | 0.227 | P | O |
| 2 | 814 | heifers | 62 | 63 | 0.026 | P | O |
| 2 | 814 | heifers | 69 | 70 | 0.024 | O | O |
| 2 | 814 | heifers | . | . | . | O | O |
| 2 | 820 | heifers | 8 | 14 | 3.158 | P | P |
| 2 | 820 | heifers | 15 | 21 | 1.5395 | P | P |
| 2 | 820 | heifers | 22 | 28 | 1.2785 | P | P |
| 2 | 820 | heifers | 29 | 35 | 0.6715 | P | P |
| 2 | 820 | heifers | 38 | 42 | 0.313 | P | P |
| 2 | 820 | heifers | 50 | 56 | 0.0725 | P | O |
| 2 | 820 | heifers | 57 | 63 | 0.039 | O | O |
| 2 | 821 | heifers | 12 | 14 | 3.272 | P | P |
| 2 | 821 | heifers | 20 | 21 | 2.2945 | P | P |
| 2 | 821 | heifers | 26 | 28 | 1.396 | P | P |
| 2 | 821 | heifers | 33 | 35 | 0.4885 | P | O |
| 2 | 821 | heifers | 40 | 42 | 0.295 | P | O |
| 2 | 821 | heifers | 47 | 49 | 0.1145 | P | O |
| 2 | 821 | heifers | 54 | 56 | 0.0585 | P | O |
| 2 | 821 | heifers | 61 | 63 | 0.0275 | P | O |
| 2 | 821 | heifers | 68 | 70 | 0.0375 | O | O |
| 2 | 821 | heifers | 75 | 77 | 0.0205 | O | O |
| 2 | 828 | heifers | 2 | 7 | 3.774 | P | P |
| 2 | 828 | heifers | 9 | 14 | 2.9965 | P | P |
| 2 | 828 | heifers | 16 | 21 | 1.631 | P | P |
| 2 | 828 | heifers | 23 | 28 | 1.186 | P | P |
| 2 | 828 | heifers | 30 | 35 | 0.035 | P | P |
| 2 | 828 | heifers | 39 | 42 | 0.232 | O | O |
| 2 | 828 | heifers | 51 | 56 | 0.0675 | O | O |
| 2 | 828 | heifers | 58 | 63 | 0.0295 | O | O |
| 2 | 835 | heifers | 17 | 21 | 2.8035 | P | P |
| 2 | 835 | heifers | 25 | 28 | 1.37 | P | P |
| 2 | 835 | heifers | 31 | 35 | 0.7795 | P | O |
| 2 | 835 | heifers | 38 | 42 | 0.2445 | P | O |
| 2 | 835 | heifers | 45 | 49 | 0.1475 | P | O |
| 2 | 835 | heifers | 52 | 56 | 0.069 | P | O |
| 2 | 835 | heifers | 59 | 63 | 0.0375 | P | O |
| 2 | 835 | heifers | 66 | 70 | 0.019 | O | O |
| 2 | 835 | heifers | 73 | 77 | 0.0325 | O | O |
| 2 | 835 | heifers | 80 | 84 | 0.6525 | O | O |
| 2 | 837 | heifers | 14 | 14 | 2.5235 | P | P |
| 2 | 837 | heifers | 22 | 28 | 1.2765 | P | P |
| 2 | 837 | heifers | 28 | 28 | 1.7025 | P | P |
| 2 | 837 | heifers | 35 | 35 | 0.245 | P | O |
| 2 | 837 | heifers | 42 | 42 | 0.141 | P | O |
| 2 | 837 | heifers | 49 | 49 | 0.0455 | P | O |
| 2 | 837 | heifers | 56 | 56 | 0.037 | P | O |
| 2 | 837 | heifers | 63 | 63 | 0.019 | O | O |
| 2 | 837 | heifers | 70 | 70 | 0.0285 | O | O |
| 2 | 837 | heifers | 77 | 77 | 0.018 | O | O |
| 2 | 840 | heifers | 15 | 21 | 2.9165 | P | P |
| 2 | 840 | heifers | 23 | 28 | 1.467 | P | P |
| 2 | 840 | heifers | 29 | 35 | 0.9735 | P | P |
| 2 | 840 | heifers | 36 | 42 | 0.2885 | P | O |
| 2 | 840 | heifers | 43 | 49 | 0.1785 | P | O |
| 2 | 840 | heifers | 50 | 56 | 0.057 | P | O |
| 2 | 840 | heifers | 57 | 63 | 0.036 | P | O |
| 2 | 840 | heifers | 64 | 70 | 0.016 | P | O |
| 2 | 840 | heifers | 71 | 77 | 0.0285 | O | O |
| 2 | 840 | heifers | 78 | 84 | 0.0225 | O | O |
| 2 | 843 | heifers | 21 | 21 | 1.853 | P | P |
| 2 | 843 | heifers | 29 | 35 | 0.793 | P | O |
| 2 | 843 | heifers | 35 | 35 | 0.521 | P | O |
| 2 | 843 | heifers | 42 | 42 | 0.105 | P | O |
| 2 | 843 | heifers | 49 | 49 | 0.0755 | P | O |
| 2 | 843 | heifers | 56 | 56 | 0.0475 | P | O |
| 2 | 843 | heifers | 63 | 63 | 0.047 | O | O |
| 2 | 843 | heifers | 70 | 70 | 0.026 | O | O |
| 2 | 843 | heifers | 77 | 77 | 0.0445 | O | O |
| 2 | 843 | heifers | 84 | 84 | 0.0215 | O | O |
| 2 | 847 | heifers | 10 | 14 | 3.141 | P | P |
| 2 | 847 | heifers | 18 | 21 | 2.389 | P | P |
| 2 | 847 | heifers | 24 | 28 | 1.2815 | P | P |
| 2 | 847 | heifers | 31 | 35 | 0.5155 | P | O |
| 2 | 847 | heifers | 38 | 42 | 0.35 | P | O |
| 2 | 847 | heifers | 45 | 49 | 0.1285 | P | O |
| 2 | 847 | heifers | 52 | 56 | 0.0735 | P | O |
| 2 | 847 | heifers | 59 | 63 | 0.0305 | O | O |
| 2 | 847 | heifers | 66 | 70 | 0.031 | O | O |
| 2 | 847 | heifers | 73 | 77 | 0.024 | O | O |
| 2 | 848 | heifers | 15 | 21 | 2.617 | P | P |
| 2 | 848 | heifers | 23 | 28 | 1.1895 | P | P |
| 2 | 848 | heifers | 29 | 35 | 0.6405 | P | O |
| 2 | 848 | heifers | 36 | 42 | 0.196 | P | O |
| 2 | 848 | heifers | 43 | 49 | 0.127 | P | O |
| 2 | 848 | heifers | 50 | 56 | 0.0835 | P | O |
| 2 | 848 | heifers | 57 | 63 | 0.0405 | O | O |
| 2 | 848 | heifers | 64 | 70 | 0.021 | O | O |
| 2 | 848 | heifers | 71 | 77 | 0.03 | O | O |
| 2 | 848 | heifers | 78 | 84 | 0.0265 | O | O |
| 2 | 852 | heifers | 18 | 21 | 2.425 | . | P |
| 2 | 852 | heifers | 26 | 28 | 1.632 | P | P |
| 2 | 852 | heifers | 32 | 35 | 0.949 | P | P |
| 2 | 852 | heifers | 39 | 42 | 0.38 | P | O |
| 2 | 852 | heifers | 46 | 49 | 0.316 | P | O |
| 2 | 852 | heifers | 53 | 56 | 0.1585 | P | O |
| 2 | 852 | heifers | 60 | 63 | 0.065 | P | O |
| 2 | 852 | heifers | 67 | 70 | 0.029 | P | O |
| 2 | 852 | heifers | 74 | 77 | 0.035 | O | O |
| 2 | 852 | heifers | 81 | 84 | 0.0235 | O | O |
| 2 | 853 | heifers | 16 | 21 | 2.091 | P | P |
| 2 | 853 | heifers | 24 | 28 | 1.2825 | P | P |
| 2 | 853 | heifers | 30 | 35 | 0.7225 | P | P |
| 2 | 853 | heifers | 37 | 42 | 0.2545 | P | P |
| 2 | 853 | heifers | 44 | 49 | 0.1715 | P | O |
| 2 | 853 | heifers | 51 | 56 | 0.1235 | P | O |
| 2 | 853 | heifers | 58 | 63 | 0.074 | P | O |
| 2 | 853 | heifers | 65 | 70 | 0.03 | O | O |
| 2 | 853 | heifers | 72 | 77 | 0.043 | O | O |
| 2 | 853 | heifers | 79 | 84 | 0.0375 | O | O |
| 2 | 855 | heifers | 13 | 14 | 3.2685 | . | P |
| 2 | 855 | heifers | 21 | 21 | 1.9755 | P | P |
| 2 | 855 | heifers | 27 | 28 | 1.155 | P | P |
| 2 | 855 | heifers | 34 | 35 | 0.3865 | P | O |
| 2 | 855 | heifers | 41 | 42 | 0.245 | P | O |
| 2 | 855 | heifers | 48 | 49 | 0.145 | P | O |
| 2 | 855 | heifers | 55 | 56 | 0.0575 | P | O |
| 2 | 855 | heifers | 62 | 63 | 0.0215 | O | O |
| 2 | 855 | heifers | 69 | 70 | 0.03 | O | O |
| 2 | 855 | heifers | 76 | 77 | 0.0195 | O | O |
| 2 | 860 | heifers | 13 | 14 | 2.6165 | P | P |
| 2 | 860 | heifers | 30 | 35 | . | P | P |
| 2 | 861 | heifers | 14 | 14 | 3.2745 | P | P |
| 2 | 861 | heifers | 22 | 28 | 2.0615 | P | P |
| 2 | 861 | heifers | 28 | 28 | 1.3775 | P | P |
| 2 | 861 | heifers | 35 | 35 | 0.357 | P | O |
| 2 | 861 | heifers | 42 | 42 | 0.2815 | P | O |
| 2 | 861 | heifers | 49 | 49 | 0.162 | P | O |
| 2 | 861 | heifers | 56 | 56 | 0.0595 | P | O |
| 2 | 861 | heifers | 63 | 63 | 0.036 | P | O |
| 2 | 861 | heifers | 70 | 70 | 0.057 | O | O |
| 2 | 861 | heifers | 77 | 77 | 0.033 | O | O |
| 2 | 862 | heifers | 2 | 7 | 3.4295 | P | P |
| 2 | 862 | heifers | 9 | 14 | 3.0075 | P | P |
| 2 | 862 | heifers | 16 | 21 | 2.4525 | P | P |
| 2 | 862 | heifers | 23 | 28 | 0.623 | P | P |
| 2 | 862 | heifers | 30 | 35 | 0.308 | P | P |
| 2 | 862 | heifers | 37 | 42 | 0.31 | P | P |
| 2 | 862 | heifers | 44 | 49 | 0.029 | P | O |
| 2 | 862 | heifers | 53 | 56 | 0.0585 | P | O |
| 2 | 862 | heifers | 65 | 70 | 0.035 | O | O |
| 2 | 862 | heifers | 72 | 77 | 0.0195 | O | O |
| 2 | 863 | heifers | 5 | 7 | 3.448 | P | P |
| 2 | 863 | heifers | 12 | 14 | 2.8685 | P | P |
| 2 | 863 | heifers | 19 | 21 | 2.441 | P | P |
| 2 | 863 | heifers | 26 | 28 | 0.7355 | P | P |
| 2 | 863 | heifers | 33 | 35 | 0.341 | P | P |
| 2 | 863 | heifers | 40 | 42 | 0.2705 | P | O |
| 2 | 863 | heifers | 47 | 49 | 0.133 | P | O |
| 2 | 863 | heifers | 56 | 56 | 0.052 | O | O |
| 2 | 863 | heifers | 68 | 70 | 0.026 | O | O |
| 2 | 863 | heifers | 75 | 77 | 0.02 | O | O |
| 2 | 864 | heifers | 19 | 21 | 1.703 | P | P |
| 2 | 864 | heifers | 27 | 28 | 0.7995 | P | P |
| 2 | 864 | heifers | 33 | 35 | 0.5515 | P | O |
| 2 | 864 | heifers | 40 | 42 | 0.1245 | P | O |
| 2 | 864 | heifers | 47 | 49 | 0.0885 | P | O |
| 2 | 864 | heifers | 54 | 56 | 0.0555 | P | O |
| 2 | 864 | heifers | 61 | 63 | 0.0295 | O | O |
| 2 | 864 | heifers | 68 | 70 | 0.0245 | O | O |
| 2 | 864 | heifers | 75 | 77 | 0.026 | O | O |
| 2 | 864 | heifers | 82 | 84 | 0.0235 | O | O |
| 2 | 865 | heifers | 12 | 14 | 2.9665 | P | P |
| 2 | 865 | heifers | 20 | 21 | 2.0275 | P | P |
| 2 | 865 | heifers | 26 | 28 | 1.618 | P | P |
| 2 | 865 | heifers | 33 | 35 | 0.382 | P | O |
| 2 | 865 | heifers | 40 | 42 | 0.165 | P | O |
| 2 | 865 | heifers | 47 | 49 | 0.161 | P | O |
| 2 | 865 | heifers | 54 | 56 | 0.05 | P | O |
| 2 | 865 | heifers | 61 | 63 | 0.0315 | O | O |
| 2 | 865 | heifers | 68 | 70 | 0.0285 | O | O |
| 2 | 865 | heifers | 75 | 77 | 0.0155 | O | O |
| 2 | 871 | heifers | 4 | 7 | 3.6325 | P | P |
| 2 | 871 | heifers | 11 | 14 | 3.11 | P | P |
| 2 | 871 | heifers | 18 | 21 | 2.672 | P | P |
| 2 | 871 | heifers | 25 | 28 | 0.9035 | P | P |
| 2 | 871 | heifers | 32 | 35 | 0.7065 | P | P |
| 2 | 871 | heifers | 39 | 42 | 0.452 | P | O |
| 2 | 871 | heifers | 46 | 49 | 0.182 | P | O |
| 2 | 871 | heifers | 55 | 56 | 0.0875 | O | O |
| 2 | 871 | heifers | 67 | 70 | 0.0405 | O | O |
| 2 | 871 | heifers | 74 | 77 | 0.0225 | O | O |
| 2 | 875 | heifers | 17 | 21 | 1.595 | P | P |
| 2 | 875 | heifers | 25 | 28 | 0.786 | P | O |
| 2 | 875 | heifers | 31 | 35 | 0.344 | P | O |
| 2 | 875 | heifers | 38 | 42 | 0.149 | P | O |
| 2 | 875 | heifers | 45 | 49 | 0.084 | P | O |
| 2 | 875 | heifers | 52 | 56 | 0.0565 | P | O |
| 2 | 875 | heifers | 59 | 63 | 0.028 | O | O |
| 2 | 875 | heifers | 66 | 70 | 0.029 | O | O |
| 2 | 875 | heifers | 73 | 77 | 0.0385 | O | O |
| 2 | 875 | heifers | 80 | 84 | 0.0215 | O | O |
| 2 | 879 | heifers | 19 | 21 | 1.54 | P | P |
| 2 | 879 | heifers | 27 | 28 | 0.668 | P | O |
| 2 | 879 | heifers | 33 | 35 | 0.4115 | P | O |
| 2 | 879 | heifers | 40 | 42 | 0.1305 | P | O |
| 2 | 879 | heifers | 47 | 49 | 0.077 | P | O |
| 2 | 879 | heifers | 54 | 56 | 0.0725 | P | O |
| 2 | 879 | heifers | 61 | 63 | 0.0355 | O | O |
| 2 | 879 | heifers | 68 | 70 | 0.0305 | O | O |
| 2 | 879 | heifers | 75 | 77 | 0.0375 | O | O |
| 2 | 879 | heifers | 82 | 84 | 0.026 | O | O |
| 2 | 881 | heifers | 11 | 14 | 2.3415 | P | P |
| 2 | 881 | heifers | 19 | 21 | 1.1435 | P | P |
| 2 | 881 | heifers | 25 | 28 | 0.5885 | P | P |
| 2 | 881 | heifers | 32 | 35 | 0.377 | P | O |
| 2 | 881 | heifers | 39 | 42 | 0.1595 | P | O |
| 2 | 881 | heifers | 46 | 49 | 0.0745 | P | P |
| 2 | 881 | heifers | 53 | 56 | 0.029 | P | O |
| 2 | 881 | heifers | 60 | 63 | 0.0265 | P | O |
| 2 | 881 | heifers | 67 | 70 | 0.0305 | P | O |
| 2 | 881 | heifers | 74 | 77 | 0.0135 | O | O |
| 2 | 882 | heifers | 8 | 14 | 3.3855 | P | P |
| 2 | 882 | heifers | 16 | 21 | 1.802 | P | P |
| 2 | 882 | heifers | 22 | 28 | 1.187 | P | P |
| 2 | 882 | heifers | 29 | 35 | 0.359 | P | O |
| 2 | 882 | heifers | 36 | 42 | 0.2445 | P | O |
| 2 | 882 | heifers | 43 | 49 | 0.1185 | P | O |
| 2 | 882 | heifers | 50 | 56 | 0.05 | P | O |
| 2 | 882 | heifers | 57 | 63 | 0.04 | O | O |
| 2 | 882 | heifers | 64 | 70 | 0.0355 | O | O |
| 2 | 882 | heifers | 71 | 77 | 0.014 | O | O |
| 2 | 884 | heifers | 17 | 21 | 1.469 | P | P |
| 2 | 884 | heifers | 25 | 28 | 0.7755 | P | O |
| 2 | 884 | heifers | 31 | 35 | 0.3355 | P | O |
| 2 | 884 | heifers | 38 | 42 | 0.121 | P | O |
| 2 | 884 | heifers | 45 | 49 | 0.0705 | P | O |
| 2 | 884 | heifers | 52 | 56 | 0.0625 | P | O |
| 2 | 884 | heifers | 59 | 63 | 0.022 | P | O |
| 2 | 884 | heifers | 66 | 70 | 0.0205 | O | O |
| 2 | 884 | heifers | 73 | 77 | 0.022 | O | O |
| 2 | 884 | heifers | 80 | 84 | 0.014 | O | O |
| 2 | 886 | heifers | 12 | 14 | 3.271 | P | P |
| 2 | 886 | heifers | 20 | 21 | 2.087 | P | P |
| 2 | 886 | heifers | 26 | 28 | 1.344 | P | P |
| 2 | 886 | heifers | 33 | 35 | 0.369 | P | P |
| 2 | 886 | heifers | 40 | 42 | 0.2125 | P | O |
| 2 | 886 | heifers | 47 | 49 | 0.15 | P | O |
| 2 | 886 | heifers | 54 | 56 | 0.053 | P | O |
| 2 | 886 | heifers | 61 | 63 | 0.031 | O | O |
| 2 | 886 | heifers | 68 | 70 | 0.0265 | O | O |
| 2 | 886 | heifers | 75 | 77 | 0.0165 | O | O |
| 2 | 887 | heifers | 12 | 14 | 2.4535 | P | P |
| 2 | 887 | heifers | 20 | 21 | 1.083 | P | P |
| 2 | 887 | heifers | 26 | 28 | 0.682 | P | O |
| 2 | 887 | heifers | 33 | 35 | 0.182 | P | P |
| 2 | 887 | heifers | 40 | 42 | 0.124 | P | O |
| 2 | 887 | heifers | 47 | 49 | 0.0725 | P | O |
| 2 | 887 | heifers | 54 | 56 | 0.04 | P | O |
| 2 | 887 | heifers | 61 | 63 | 0.029 | O | O |
| 2 | 887 | heifers | 68 | 70 | 0.027 | P | O |
| 2 | 887 | heifers | 75 | 77 | 0.017 | P | O |
| 2 | 890 | heifers | 16 | 21 | 2.5065 | P | P |
| 2 | 890 | heifers | 24 | 28 | 1.118 | P | P |
| 2 | 890 | heifers | 30 | 35 | 0.7915 | P | P |
| 2 | 890 | heifers | 37 | 42 | 0.226 | P | O |
| 2 | 890 | heifers | 44 | 49 | 0.19 | P | O |
| 2 | 890 | heifers | 51 | 56 | 0.1225 | P | O |
| 2 | 890 | heifers | 58 | 63 | 0.039 | P | O |
| 2 | 890 | heifers | 65 | 70 | 0.0375 | P | O |
| 2 | 890 | heifers | 72 | 77 | 0.0355 | P | O |
| 2 | 890 | heifers | 79 | 84 | 0.021 | P | O |
| 2 | 891 | heifers | 17 | 21 | 2.056 | P | P |
| 2 | 891 | heifers | 25 | 28 | 0.7805 | P | O |
| 2 | 891 | heifers | 31 | 35 | 0.5575 | P | P |
| 2 | 891 | heifers | 38 | 42 | 0.166 | P | O |
| 2 | 891 | heifers | 45 | 49 | 0.1305 | P | O |
| 2 | 891 | heifers | 52 | 56 | 0.1 | P | O |
| 2 | 891 | heifers | 59 | 63 | 0.037 | O | O |
| 2 | 891 | heifers | 66 | 70 | 0.04 | O | O |
| 2 | 891 | heifers | 73 | 77 | 0.0485 | O | O |
| 2 | 891 | heifers | 80 | 84 | 0.0255 | O | P |
| 2 | 896 | heifers | 1 | 7 | 3.691 | . | . |
| 2 | 896 | heifers | 8 | 14 | 2.9195 | P | P |
| 2 | 896 | heifers | 15 | 21 | 2.22 | P | P |
| 2 | 896 | heifers | 22 | 28 | 0.6385 | P | P |
| 2 | 896 | heifers | 29 | 35 | 0.5785 | P | P |
| 2 | 896 | heifers | 36 | 42 | 0.3125 | P | O |
| 2 | 896 | heifers | 43 | 49 | 0.1065 | P | O |
| 2 | 896 | heifers | 52 | 56 | 0.0455 | P | O |
| 2 | 896 | heifers | 64 | 70 | 0.0255 | O | O |
| 2 | 896 | heifers | 71 | 77 | 0.0195 | O | O |
| 2 | 898 | heifers | 10 | 14 | 3.529 | P | P |
| 2 | 898 | heifers | 18 | 21 | 2.541 | P | P |
| 2 | 898 | heifers | 24 | 28 | 1.6645 | P | P |
| 2 | 898 | heifers | 31 | 35 | 0.5385 | P | O |
| 2 | 898 | heifers | 38 | 42 | 0.365 | P | O |
| 2 | 898 | heifers | 45 | 49 | 0.215 | P | O |
| 2 | 898 | heifers | 52 | 56 | 0.0635 | O | O |
| 2 | 898 | heifers | 59 | 63 | 0.0475 | O | O |
| 2 | 898 | heifers | 66 | 70 | 0.0385 | O | O |
| 2 | 898 | heifers | 73 | 77 | 0.0195 | O | O |
| 1 | 4114 | cows | 4 | 7 | . | P | P |
| 1 | 4114 | cows | 12 | 14 | 2.3335 | P | P |
| 1 | 4114 | cows | 18 | 21 | 1.5975 | P | P |
| 1 | 4114 | cows | 25 | 28 | 0.5465 | P | P |
| 1 | 4114 | cows | 32 | 35 | 0.315 | P | P |
| 1 | 4114 | cows | 39 | 42 | 0.1335 | P | O |
| 1 | 4114 | cows | 46 | 49 | 0.102 | P | O |
| 1 | 4114 | cows | 53 | 56 | 0.0465 | P | O |
| 1 | 4114 | cows | 60 | 63 | 0.036 | P | O |
| 1 | 4114 | cows | 67 | 70 | 0.025 | O | O |
| 1 | 4114 | cows | 76 | 77 | 0.024 | P | O |
| 1 | 5103 | cows | 2 | 7 | 3.765 | P | P |
| 1 | 5103 | cows | 8 | 14 | 3.558 | P | P |
| 1 | 5103 | cows | 15 | 21 | 2.266 | P | P |
| 1 | 5103 | cows | 22 | 28 | 1.834 | P | P |
| 1 | 5103 | cows | 29 | 35 | 0.7455 | P | P |
| 1 | 5103 | cows | 36 | 42 | 0.6345 | P | P |
| 1 | 5103 | cows | 43 | 49 | 0.1895 | P | P |
| 1 | 5103 | cows | 50 | 56 | 0.1615 | P | O |
| 1 | 5103 | cows | 57 | 63 | 0.103 | P | O |
| 1 | 5103 | cows | 66 | 70 | 0.047 | O | O |
| 1 | 6111 | cows | 9 | 14 | 3.247 | P | P |
| 1 | 6111 | cows | 15 | 21 | 2.8875 | P | P |
| 1 | 6111 | cows | 22 | 28 | 1.451 | P | P |
| 1 | 6111 | cows | 29 | 35 | 0.6885 | P | P |
| 1 | 6111 | cows | 36 | 42 | 0.356 | P | O |
| 1 | 6111 | cows | 43 | 49 | 0.2245 | P | P |
| 1 | 6111 | cows | 50 | 56 | 0.085 | P | O |
| 1 | 6111 | cows | 57 | 63 | 0.0605 | P | O |
| 1 | 6111 | cows | 64 | 70 | 0.042 | P | O |
| 1 | 6111 | cows | 73 | 77 | 0.024 | P | O |
| 1 | 7104 | cows | 13 | 14 | 2.012 | P | P |
| 1 | 7104 | cows | 19 | 21 | 1.7025 | P | P |
| 1 | 7104 | cows | 26 | 28 | 0.619 | P | P |
| 1 | 7104 | cows | 33 | 35 | 0.2275 | P | O |
| 1 | 7104 | cows | 40 | 42 | 0.1255 | P | O |
| 1 | 7104 | cows | 47 | 49 | 0.0775 | P | O |
| 1 | 7104 | cows | 54 | 56 | 0.0495 | P | O |
| 1 | 7104 | cows | 61 | 63 | 0.0335 | O | O |
| 1 | 7104 | cows | 68 | 70 | 0.075 | O | O |
| 1 | 7104 | cows | 77 | 77 | 0.072 | O | O |
| 1 | 7116 | cows | 5 | 7 | 3.5635 | P | P |
| 1 | 7116 | cows | 11 | 14 | 2.918 | P | P |
| 1 | 7116 | cows | 18 | 21 | 1.304 | P | P |
| 1 | 7116 | cows | 25 | 28 | 0.7375 | P | P |
| 1 | 7116 | cows | 32 | 35 | 0.336 | P | O |
| 1 | 7116 | cows | 39 | 42 | 0.201 | P | P |
| 1 | 7116 | cows | 46 | 49 | 0.0815 | P | O |
| 1 | 7116 | cows | 53 | 56 | 0.063 | O | O |
| 1 | 7116 | cows | 60 | 63 | 0.0905 | P | O |
| 1 | 7116 | cows | 69 | 70 | 0.056 | O | O |
| 1 | 7116 | cows | 78 | 84 | 0.056 | O | O |
| 1 | 7116 | cows | 88 | 84 | 0.0475 | O | O |
| 1 | 8104 | cows | 6 | 7 | 2.9075 | P | P |
| 1 | 8104 | cows | 13 | 14 | 2.359 | P | P |
| 1 | 8104 | cows | 20 | 21 | 1.863 | P | O |
| 1 | 8104 | cows | 27 | 28 | 0.468 | P | . |
| 1 | 8104 | cows | 34 | 35 | 0.4205 | P | P |
| 1 | 8104 | cows | 41 | 42 | 0.2165 | P | O |
| 2 | 8104 | heifers | 46 | 49 | 0.468 | P | P |
| 1 | 8104 | cows | 48 | 49 | 0.088 | P | O |
| 1 | 8104 | cows | 57 | 63 | 0.051 | P | O |
| 1 | 8104 | cows | 69 | 70 | 0.0295 | O | O |
| 1 | 8104 | cows | 76 | 77 | 0.0205 | O | O |
| 1 | 8106 | cows | 6 | 7 | 2.7035 | P | P |
| 1 | 8106 | cows | 14 | 14 | 1.142 | P | O |
| 1 | 8106 | cows | 20 | 21 | 0.819 | P | O |
| 1 | 8106 | cows | 27 | 28 | 0.185 | P | O |
| 1 | 8106 | cows | 34 | 35 | 0.132 | P | O |
| 1 | 8106 | cows | 41 | 42 | 0.099 | P | O |
| 2 | 8106 | heifers | 46 | 49 | 0.033 | P | O |
| 1 | 8106 | cows | 48 | 49 | 0.033 | P | . |
| 1 | 8106 | cows | 55 | 56 | 0.028 | O | O |
| 1 | 8106 | cows | 62 | 63 | 0.027 | O | O |
| 1 | 8106 | cows | 69 | 70 | 0.018 | O | O |
| 1 | 8106 | cows | 78 | 84 | 0.0195 | O | O |
| 2 | 8114 | heifers | 15 | 21 | 1.2065 | P | P |
| 2 | 8114 | heifers | 23 | 28 | 0.4435 | P | O |
| 2 | 8114 | heifers | 29 | 35 | 0.3205 | P | O |
| 2 | 8114 | heifers | 36 | 42 | 0.061 | P | O |
| 2 | 8114 | heifers | 43 | 49 | 0.0595 | P | O |
| 2 | 8114 | heifers | 50 | 56 | 0.042 | P | O |
| 2 | 8114 | heifers | 57 | 63 | 0.0205 | O | O |
| 2 | 8114 | heifers | 64 | 70 | 0.0165 | O | O |
| 2 | 8114 | heifers | 71 | 77 | 0.0195 | O | O |
| 2 | 8114 | heifers | 78 | 84 | 0.0225 | O | O |
| 2 | 8116 | heifers | 13 | 14 | 3.4325 | P | P |
| 2 | 8116 | heifers | 21 | 21 | 2.3925 | P | P |
| 2 | 8116 | heifers | 27 | 28 | 1.513 | P | P |
| 2 | 8116 | heifers | 34 | 35 | 0.4575 | P | O |
| 2 | 8116 | heifers | 41 | 42 | 0.342 | P | O |
| 2 | 8116 | heifers | 48 | 49 | 0.205 | P | O |
| 2 | 8116 | heifers | 55 | 56 | 0.0715 | O | O |
| 2 | 8116 | heifers | 62 | 63 | 0.047 | O | O |
| 2 | 8116 | heifers | 69 | 70 | 0.0415 | O | O |
| 2 | 8116 | heifers | 76 | 77 | 0.0245 | O | O |
| 2 | 8118 | heifers | 25 | 28 | 0.554 | P | . |
| 2 | 8118 | heifers | 57 | 63 | 0.0375 | P | . |
| 2 | 8118 | heifers | 57 | 63 | 0.0375 | P | . |
| 2 | 8123 | heifers | 21 | 21 | 2.2615 | P | P |
| 2 | 8123 | heifers | 29 | 35 | 2.1715 | P | P |
| 2 | 8123 | heifers | 35 | 35 | 0.6195 | P | O |
| 2 | 8123 | heifers | 42 | 42 | 0.1475 | P | O |
| 2 | 8123 | heifers | 49 | 49 | 0.1 | P | O |
| 2 | 8123 | heifers | 56 | 56 | 0.074 | P | O |
| 2 | 8123 | heifers | 63 | 63 | 0.0345 | O | O |
| 2 | 8123 | heifers | 70 | 70 | 0.0265 | O | O |
| 2 | 8123 | heifers | 77 | 77 | 0.031 | O | O |
| 2 | 8123 | heifers | 84 | 84 | 0.022 | O | O |
| 2 | 8126 | heifers | 15 | 21 | 2.791 | P | P |
| 2 | 8126 | heifers | 23 | 28 | 1.1715 | P | P |
| 2 | 8126 | heifers | 29 | 35 | 1.2945 | P | P |
| 2 | 8126 | heifers | 36 | 42 | 0.194 | P | O |
| 2 | 8126 | heifers | 43 | 49 | 0.1305 | P | O |
| 2 | 8126 | heifers | 50 | 56 | 0.092 | P | O |
| 2 | 8137 | heifers | 8 | 14 | 3.6275 | P | P |
| 2 | 8137 | heifers | 16 | 21 | 2.7375 | P | P |
| 2 | 8137 | heifers | 22 | 28 | 1.915 | P | P |
| 2 | 8137 | heifers | 29 | 35 | 0.654 | P | P |
| 2 | 8137 | heifers | 36 | 42 | 0.3775 | P | O |
| 2 | 8137 | heifers | 43 | 49 | 0.2095 | P | O |
| 2 | 8137 | heifers | 50 | 56 | 0.087 | O | O |
| 2 | 8137 | heifers | 57 | 63 | 0.0515 | O | O |
| 2 | 8137 | heifers | 64 | 70 | 0.054 | O | O |
| 2 | 8137 | heifers | 71 | 77 | 0.0315 | O | O |
| 2 | 8137 | heifers | 80 | 84 | 0.0435 | . | . |
| 2 | 8143 | heifers | 7 | 7 | 2.9955 | P | P |
| 2 | 8143 | heifers | 14 | 14 | 1.968 | P | P |
| 2 | 8143 | heifers | 21 | 21 | 1.364 | P | P |
| 2 | 8143 | heifers | 28 | 28 | 0.493 | P | P |
| 2 | 8143 | heifers | 35 | 35 | 0.266 | P | P |
| 2 | 8143 | heifers | 42 | 42 | 0.1675 | P | O |
| 2 | 8143 | heifers | 49 | 49 | 0.055 | O | O |
| 2 | 8143 | heifers | 70 | 70 | 0.024 | O | O |
| 2 | 8143 | heifers | 77 | 77 | 0.024 | O | O |
| 2 | 8143 | heifers | . | . | . | O | O |
| 2 | 8148 | heifers | 14 | 14 | 3.192 | P | P |
| 2 | 8148 | heifers | 22 | 28 | 2.044 | P | P |
| 2 | 8148 | heifers | 28 | 28 | 0.8625 | P | P |
| 2 | 8148 | heifers | 35 | 42 | 0.297 | P | O |
| 2 | 8148 | heifers | 42 | 42 | 0.192 | P | O |
| 2 | 8148 | heifers | 49 | 49 | 0.1385 | P | O |
| 2 | 8148 | heifers | 56 | 56 | 0.0805 | O | O |
| 2 | 8148 | heifers | 63 | 63 | 0.1215 | P | O |
| 2 | 8148 | heifers | 70 | 70 | 0.1065 | O | O |
| 2 | 8148 | heifers | 77 | 77 | 0.0595 | O | O |
| 1 | 840U | cows | 7 | 7 | 3.3315 | P | P |
| 1 | 840U | cows | 13 | 14 | 2.4135 | P | P |
| 1 | 840U | cows | 20 | 21 | 1.2505 | P | P |
| 1 | 840U | cows | 27 | 28 | 0.68 | P | P |
| 1 | 840U | cows | 34 | 35 | 0.231 | P | P |
| 1 | 840U | cows | 41 | 42 | 0.1385 | P | O |
| 1 | 840U | cows | 48 | 49 | 0.0555 | P | O |
| 1 | 840U | cows | 55 | 56 | 0.0415 | P | O |
| 1 | 840U | cows | 62 | 63 | 0.024 | P | O |
| 1 | 840U | cows | 71 | 77 | 0.0175 | O | O |
