# Supplementary material for: Evaluation of pregnancy associated glycoproteins assays for on farm determination of pregnancy status in beef cattle
Source: PLoS One. 2024 Jul 25;19(7):e0306325. doi: 10.1371/journal.pone.0306325 (PMC11271854; doi:10.1371/journal.pone.0306325)
Supplement: Attachment — Submitted filename: PONE-D-24-00715_reviewer comments.docx [file pone.0306325.s004.docx]

Overall, the manuscript is well written and easy to read. It details an evaluation of a newly marketed cow-side pregnancy test. Evaluation of such new tools is important for the beef and dairy industry including veterinarians and producers. Understanding test function, limitations, and accuracy is critically important for those making decisions on whether to use such tools for their herd. There is certainly value in reporting the results of this study. Below are some items to consider to strengthen the manuscript as currently written.

Lines 59-61 – Beef NAHMS 2017 survey from USDA has data on the percentage of beef herds that use ultrasound for pregnancy diagnosis or perform any pregnancy diagnosis at all. According to this data set, only 31.6% of beef operations surveyed used either blood based, ultrasound, or palpation pregnancy diagnosis. Including this data could strengthen your argument for use of a rapid, cow-side test for pregnancy and the impacts on production efficiency and profitability.

Line 71 – Roberts et al., *Reprod Domest Anim* 2015; 50(4):651-8 investigated the use of a milk-based ELISA in beef cows. This could be included here to indicate it’s not only been investigated in dairy cows.

The ultimate gold standard for comparison of any pregnancy diagnosis method is confirmed delivery of a fetus. Since this study spanned 3 years, do you have calving data for which you would be able to compare and calculate sensitivity and specificity of the pregnancy diagnosis method used relative to calving? This would also allow for some discussion of rates of loss of pregnancy in beef cows which would be valuable to practitioners and producers.

Reporting the loss rates between the 28d ultrasound examination and the recheck, as well as how this compared to the results of the assays would be useful and practical information for users of these tests.

Lines 145-147 – We intra- and inter-assay coefficients of variation calculated for the OD readings on the RPT? What were the cutoffs used for pregnant, non-pregnant, and inconclusive/recheck?

There are no sensitivities or specificities calculated in this study. You have only reported false negative and false positive results with NPV and PPV which can be impacted by the population of this one study (ie prevalence of open or pregnant cows in your study population). The sensitivity and specificity, along with confidence intervals, should be included, as this reflects the expected performance of the test. Previous studies comparing PAG tests to a gold standard, routinely report sensitivity and specificity in addition to test agreement and NPV/PPV. Please incorporate this into the manuscript.

Figures 1 and 2 do not, in this reviewer’s opinion, add additional insight into the data. While it shows a linear relationship of PAG OD to dpp for each parity group or dpp group, the following Figure 3 is a clearer depiction of the relationship of dpp OD, and assay cut off. It is this reviewer’s recommendation to remove Figures 1 and 2, keeping figure 3.

There are multiple mentions of the PAG levels “plateauing” during the postpartum period. Plateau typically indicates that something has reached its highest point and stays there but in this case, the PAGs have reached a low point. From a clarity standpoint for the reader, consider using different terminology such as “sustained nadir” or “baseline” to indicate the PAG levels have reached their lowest point and stayed there.

Lines 330-359 – There is extensive discussion of the previous studies and days at which the performance of the test was evaluated. Despite referencing Ricci, et al (Ref #10), early in the manuscript, there is no mention here of their finding that the IDEXX RPT has a period where PAG OD may drop into the recheck or inconclusive range (d 46-67 post AI) and was more pronounced in multiparous cows. Roberts et al 2015 also found this to be a gestational window where false negatives were possible in beef cows. This is of particular importance for beef producers using this test and understanding its limitations when the population they are testing may have a wide range of days of gestation depending on breeding strategy. These limitations identified by Ricci and discussed in both the Ricci and Roberts studies are hypothesized to be due to varying expression of PAG throughout early gestation. Given that the specific PAG in the lateral flow is unknown, is it possible that there could also be a window of gestation with this test that yields equivocal results or contributes to some of the false negatives depending on the expression pattern of the monoclonal PAG antibody used?

Lines 378-384 – It is unclear what the process of uterine involution has to do with the use of a test to detect PAGs. Presumably, the authors are inferring that uterine involution is a critical step in return to fertility in the postpartum beef cow prior to the next conception. However, the way it is worded in this section is confusing. Please rework this paragraph to clarify the relationship you are making between uterine involution and the use of the blood-based pregnancy testing methods for the next pregnancy.
