# Supplementary material for: Evaluation of pregnancy associated glycoproteins assays for on farm determination of pregnancy status in beef cattle
Source: PLoS One. 2024 Jul 25;19(7):e0306325. doi: 10.1371/journal.pone.0306325 (PMC11271854; doi:10.1371/journal.pone.0306325)
Supplement: Attachment — Submitted filename: response to review.docx [file pone.0306325.s006.docx]

Dr. Abuelo,

We greatly appreciate the effort you and the reviewers have made in reviewing this manuscript. We have incorporated almost all of the suggested comments, and our individual responses to each suggestion is listed below.

Line 63-65 This section, starting with “In order…” is a sentence fragment. Please revise so it is more clear and complete.

This has been revised to read “Pregnancy diagnosis within an operation is not only important, but necessary to increase profitability and have a complete and successful reproductive management program”

Line 237 – In table and in legend, please correct spelling of “sensitivity”

Revised as suggested

Lines 393-394 – In this sentence, it would be appropriate to use “ultrasound equipment” rather than “equipment of ultrasonography”

Revised as suggested


Lines 394-397 – A better way to discuss the costs would be to state that the blood test methods range from $4.50 to $8 per head while the cost for ultrasound is going to depend on the veterinarian’s rate to perform the test and their speed. Many veterinarians, working with an efficient crew, can run 30-45 cows through the chute in an hour (or more) likely making the cost of all methods relatively similar and maybe even less per head for ultrasonography since veterinarians typically charge an hourly rate for these services (rather than per head). It also needs to be mentioned that a skilled ultrasonographer can provide valuable information about the pregnancy (viability, stage of gestation, fetal sex, etc) that is not provided by a blood test. Because of the extreme variability in rates charged by practicing veterinarians providing ultrasonography services across the country, I would suggest not including that dollar figure. Additionally, the most logical argument for the use of a cow side test is in rural areas where producers are unable to find veterinarians to perform pregnancy diagnosis. This tool allows those producers to benefit from pregnancy diagnosis and identification of non-pregnant cows which will help their bottom line even when professional services are unavailable.

Revised as suggested


Lines 479-481 – The added sentences do not flow with the rest of the paragraph content. Please consider revising so they complete the paragraph. One suggested revision would be to word as follows: “Due to the additional time required for diagnosis of pregnancy with blood based tests, management decisions may be delayed compared to ultrasonography and may result in additional labor to sort open females once test results are available.”

This has been revised as suggested.
